# Assessment of myocardial function and cardiac performance using left ventricular global longitudinal strain in athletes after COVID-19: a follow-up study

**DOI:** 10.3389/fcvm.2023.1240278

**Published:** 2023-10-09

**Authors:** J. Schellenberg, L. Matits, D. A. Bizjak, J. Kersten, J. Kirsten, S. Vollrath, J. M. Steinacker

**Affiliations:** ^1^ Division of Sports and Rehabilitation Medicine, University Ulm Hospital, Ulm, Germany; ^2^ Clinical & Biological Psychology, Institute of Psychology and Education, Ulm University, Ulm, Germany

**Keywords:** sport, COVID-19, speckle tracking echocardiography, performance, CPET

## Abstract

**Background:**

It has not yet been conclusively determined whether reduced left ventricular global longitudinal strain (LV GLS) after COVID-19 contributes to a reduction in exercise capacity. Our own studies showed a possible mild myocardial involvement in the form of reduced LV GLS in athletes after COVID-19 compared with healthy athletes. The aims of this prospective follow-up study were to investigate the development of LV GLS over a 3-month period in athletes after COVID-19 and the possible relationship between LV GLS and physical performance.

**Methods:**

LV GLS was determined in four-, two-, and three-chamber views and assessed offline by a blinded investigator in 96 recreational athletes (mean age 33.15 ± 12.40 years, 53 male, peak VO_2_ 38.82 ± 11.14 ml/min/kg) at a median of two (*t*_0_) and five months (*t*_1_) after COVID-19. Cardiopulmonary exercise testing (CPET) was performed on a bicycle ergometer on both examination dates.

**Results:**

LV GLS improved significantly between *t*_0_ and *t*_1_ (*t*_0_ −18.82 ± 2.02 vs. *t*_1_ −19.46 ± 2.05, *p* < 0.001). Echocardiographic and spiroergometric parameters were within the normal clinical reference range. Maximum power increased significantly from *t*_0_ to *t*_1_ (*t*_0_ 283.17 ± 83.20 vs. *t*_1_ 286.24 ± 85.22 Watt, *p* = 0.009) and there was a trend toward increased peak oxygen uptake (*t*_0_ 36.82 ± 11.14 vs. *t*_1_ 38.68 ± 10.26 ml/min/kg, *p* = 0.069). We found no correlation between LV GLS and performance parameters, except for the respiratory exchange ratio (RER) [*ρ* −0.316, (−0.501; −0.102), *p* < 0.050].

**Conclusions:**

Significant improvement in LV GLS approximately five months after COVID-19 may be due to mild myocardial involvement during or shortly after COVID-19, which seems to recover. There was no correlation between LV GLS and performance parameters, except for an inverse correlation of LV GLS and RER, suggesting insufficient exercise intolerance at lower GLS values. Further studies on the development of GLS in athletes or in the general population with moderate and severe disease courses would be informative as well as the comparison of pre-COVID-19 with post-COVID-19 echocardiography to evaluate the effects of COVID-19 on cardiac function.

## 1. Introduction

Coronavirus Disease 2019 (COVID-19) is a systemic viral infection caused by Severe Acute Respiratory Syndrome-Coronavirus-2 (SARS-CoV-2) that primarily affects the respiratory system but can also cause myocardial damage (1–3). Studies in elite athletes have shown that the infection is often mild (46%–82%) or asymptomatic (16%–58%) (4–7), with myocarditis occurring only in rare cases (1%–3%) (8–9). Most athletes can return to competitive and amateur sports after a training break adapted to current symptoms (10–12). However, having passed through COVID-19 does not necessarily imply a complete recovery to former health or performance status and a return-to-sport examination with echocardiography should be performed especially in case of cardiac symptoms during and/or after infection (11).

Analysis of myocardial deformation by speckle-tracking echocardiography (STE) can provide additional information to the standard echocardiography examination (13). The global longitudinal strain of the left ventricle (LV GLS) is more sensitive than left ventricular ejection fraction (LV EF) alone in detecting subclinical LV dysfunction (14) and is a prognostic parameter for long-term risk of cardiovascular morbidity and mortality (15). Individual studies demonstrated reduced LV GLS with preserved ejection fraction (pEF) in the setting of acute SARS-CoV-2 infection in hospitalized patients regardless of infection severity (16–19) and in patients recovered from COVID-19 (20–23). No changes in LV GLS have been observed in athletes compared with healthy athletes 22 days (24) and 19 days (25) after COVID-19. Conversely, we demonstrated reduced LV GLS and diastolic function in a cohort of athletes at a median of two months after COVID-19 compared with non-infected healthy athletes (26).

Longitudinally, hospitalized patients showed no significant improvement in LV GLS at two months (27) or three months after acute infection (28), and 25% of patients still had abnormal LV GLS three months after acute infection (28). However, in the follow-up study by Karagodin et al., improvements in LV GLS were noted in patients with impaired baseline function (29). Further long-term observations in athletes are scarce.

The two main aims of this prospective follow-up study were, first, to investigate the development of LV GLS in athletes without history of LV dysfunction at an average of two months after SARS-CoV-2 infection to a follow-up of three months, and, second, to find if there may be a relationship between myocardial changes detected by LV GLS determination and physical performance.

## 2. Methods

### 2.1. Study population

Ninety-six recreational athletes presenting to the Ulm Clinic for Sports and Rehabilitation Medicine after COVID-19 were included in this prospective, single-center, longitudinal cohort study after being informed of the study procedures and providing written informed consent. The results presented in this study are from baseline clinical assessment between June 2020 and July 2022, a median of two months (IQR: one to five months) after COVID-19, and follow-up three months later. Study participants were included consecutively. They engaged in endurance sports, strength sports, team sports, or technical sports with a training volume of at least three times per week, corresponding to more than 20 metabolic equivalents of task (MET) per week. The weekly training time among the recreational athletes was about five to eight hours. Additional inclusion criteria were: ≥18 years of age and a positive SARS-CoV-2 PCR test or antibody detection with additional typical symptoms. The exclusion criteria were: acute or chronic illness that precluded a planned physical examination, acute SARS-CoV-2 infection, refusal of peripheral venous blood sampling, inadequate German language skills, and withdrawal from study participation. Athletes provided written informed consent after being instructed of the study procedures (30). The study was conducted in accordance with the Declaration of Helsinki and approved by the local ethics committee of the University of Ulm (EK 408/20).

### 2.2. Clinical evaluation of the participants

All athletes underwent a clinical evaluation that included a medical history and physical examination, 12-lead electrocardiogram (ECG), transthoracic echocardiography (TTE) including determination of left ventricular global longitudinal strain (LV GLS), and cardiopulmonary exercise testing (CPET). To examine the long-term course of echocardiographic and CPET findings, participants were invited for a follow-up three months after the initial clinical evaluation.

### 2.3. Transthoracic echocardiography

Transthoracic examination was performed using an EPIQ 7 ultrasound system with a phased-array probe X5-1 (Philips GmbH, Hamburg, Germany). The following parameters were collected: left ventricular internal diameter end diastole (LVIDd) and end systole (LVIDs). LVIDd (LVIDd/BSA) and LVIDs (LVIDs/BSA) were indexed to body surface area (BSA), left ventricular ejection fraction (LV-EF by biplane LV planimetry by Simpson), fractional shortening (FS), left ventricular mass (LV mass), LV mass/BSA, stroke volume (SV), septal thickness [= interventricular septal end diastole (IVSd)] and posterior wall thickness [= left ventricular posterior wall end diastole (LVPWd)] and right ventricle longitudinal function by tricuspid annular plane systolic excursion (TAPSE). Diastolic function was characterized by E/A ratio, E/Élateral ratio, E/E'medial ratio and deceleration time (Dec Time) (31, 32).

### 2.4. Strain measurements

LV GLS was determined offline using TomTec postprocessing software (2D Cardiac Performance Analysis, TomTec Imaging Systems, Unterschleissheim, Germany) by an examiner who was blinded to patient history. LV GLS was obtained in apical four-chamber, two-chamber and long-axis views in the apical, midline, and basal segments (33). The endocardial contour was manually adjusted. Regardless of provider or clinical covariates, a LV GLS ≥−16% (less negative) was considered abnormal (34). A selection of 20 images was reviewed a second time by the same blinded investigator and by a second blinded investigator to determine intrarater and interrater reliability.

### 2.5. Cardiopulmonary exercise testing (CPET)

CPET was performed on a cycle ergometer (Excalibur Sport, LODE B.V., Groningen, The Netherlands) using a breath-by-breath gas analysis system (Ergostik, Geratherm Respiratory, Bad Kissingen, Germany). All athletes performed an incremental exercise test. An individually adjusted ramp protocol was chosen according to age, gender, weight and estimated fitness level to exhaust subjects within 8–12 min (35). A 12 lead ECG recording system (Cardiopart 12 Blue/Blue-P, AMEDTEC Medizintechnik Aue GmbH, Aue, Germany) was used. All CPETs were evaluated by the same investigator according to Wasserman et al. (35) and Clinical Recommendations for Cardiopulmonary Exercise Testing Data Assessment in Specific Patient Populations (36). The following variables were measured or calculated: maximum power and predicted maximum power, respiratory oxygen uptake at first ventilatory threshold (VO_2_@VT1/kg) and peak respiratory oxygen uptake (peak VO_2_/kg), peak respiratory exchange rate (RER), heart rate (HR) at VT1 (HR@VT1), HR at peak respiratory oxygen uptake (HR@peak VO_2_) and predicted HR at peak respiratory oxygen uptake (predicted HR@peak VO_2_), peak oxygen pulse (peak O_2_/HR) and predicted peak oxygen pulse and the ventilation/volume of CO_2_ slope (VE/VCO_2_ slope).

### 2.6. Statistical analysis

Statistical analyses were performed using R Project for Statistical Computing version 4.1.1 (RRID:SCR_001905) (37) and GraphPad Prism 9 (Version 9.4.1, GraphPad Software Inc., California, USA, RRID:SCR_002798). The descriptive data are presented as median and interquartile ranges (IQR). Assumptions for linear regression were visually verified using residual, QQ plots, and histograms. For distribution analysis, Shapiro-Wilk test was used. Correlations between LV GLS and age and BMI were analyzed using Spearman's *ρ*. The change of cardiac and performance parameters over the three month period was assessed using robust linear mixed effects regression models (38) separately controlling for possible confounding variables (BMI, age, sex, HR, systolic and diastolic blood pressure). A *p*-value of <0.05 was considered significant. We performed an additional exploratory analysis in a subsample of six athletes with a GLS ≥−16%, defined as reduced GLS. Comparisons were made with a paired *t*-test if the distribution was normal, otherwise a Wilcoxon signed-rank test was used.

## 3. Results

### 3.1. Cohort characteristics

A total of 96 athletes (mean age 33.15 ± 12.40 years, 53 male) were included in the statistical analysis. The sports practiced were divided as follows: 61% endurance sports, 13% strength and combat sports, 21% team sports and 5% technical sports (Table 1). Symptoms reported during COVID-19 infection were standard clinical symptoms of viral infection: fever (39%), cough (46%), rhinorrhea (52%), sore throat (45%), resting dyspnea (21%) or exertional dyspnea (34%), and subjectively perceived reduction in performance (39%) compared with maximal performance before COVID-19. Cardiac symptoms were observed in the form of palpitations (21%), chest pain (21%), increased resting heart rate (26%) or exertional dyspnea after COVID-19 (30%) (Table 2).

**Table 1:** Demographic characteristics.

Number	96	
Sex (male/female)	53/43 (55/45%)	
	Means ± SD	Median (IQR)
Age (years)	33.15 ± 12.40	30 (22.75–40.50)
Weight (kg)	73.93 ± 14.86	71.50 (63.64–84.40)
Height (cm)	175.81 ± 9.05	175.25 (169–183.25)
BMI (kg/m2)	23.68 ± 3.51	23.05 (21.20–25.38)
BSA (g/m2)	1.89 ± 0.21	1.88 (1.72–2.06)
CK (U/l) [normal 20–180 U/l]	204.34 ± 419.69	109 (75.25–170.75)
Troponin T (ng/l) [normal <14 ng/l]	5.94 ± 4.00	4 (3–7.5)
CRP (mg/l) [normal < 0.6 mg/l]	1.10 ± 1.81	0.6 (0.6–0.9)
Ferritin (μg/l) [normal 22–112 (μg/l]	127.97 ± 111.16	86.50 (52.25–174.75)
Hemoglobin (g/dl) [normal 12.3–15.3]	14.33 ± 1.35	14.20 (13.20–15.30)
Training volume	At least three times per week; > 20 MET/week; five to eight hours/week	
Endurance sports	59 (61%)	
Running	20 (21%)	
Triathlon	11 (12%)	
Cycling	8 (8%)	
Nordic Walking	7 (7%)	
Rowing	6 (6%)	
Others	7 (7%)	
Strength sports	12 (13%)	
Team sports	20 (21%)	
Soccer	13 (14%)	
Handball	4 (4%)	
Others	3 (3%)	
Technical sports	5 (5%)	

SD, standard deviation; IQR, interquartile range; BMI, body mass index; MET, metabolic equivalents of task. Sports are differentiated according to the predominant component. Based on the 2020 ESC Guidelines on sports cardiology and exercise in patients with cardiovascular disease (39).

**Table 2:** Symptoms during COVID-19 presented as absolute values and relative frequencies.

Symptoms	Present	Not present	Missing
Fever	37 (39%)	45 (46%)	14 (15%)
Cough	45 (46%)	37 (39%)	14 (15%)
Rhinorrhea	50 (52%)	32 (33%)	14 (15%)
Sore throat	43 (45%)	39 (40%)	14 (15%)
Resting dyspnea	20 (21%)	62 (64%)	14 (15%)
Exertional dyspnea during covid-19	33 (34%)	49 (51%)	14 (15%)
Exertional dyspnea after covid-19	29 (30%)	61 (64%)	6 (6%)
Palpitations	20 (21%)	70 (73%)	6 (6%)
Chest pain	20 (21%)	70 (73%)	6 (6%)
Increased resting heart rate	25 (26%)	65 (68%)	6 (6%)
Subjective perceived performance limitation	37 (39%)	53 (55%)	6 (6%)
Dizziness	27 (28%)	63 (66%)	6 (6%)

### 3.2. Echocardiographic parameters

LV GLS improved significantly between *t*_0_ and *t*_1_ (*t*_0_ (−18.82 ± 2.02 vs. *t*_1_ −19.46 ± 2.05, *p* < 0.001). All echocardiographic parameters were within the normal range. There were no significant differences between the study time points (Table 3, Supplementary Table S1). Significant changes in LV GLS persisted over time even after adjustment for confounding variables (age, sex, heart rate, BMI, systolic or diastolic blood pressure) (Supplementary Table S2). Intrarater and interrater reliability with respect to the LV GLS measure showed high agreement (intrarater: 0.892 [95%CI, 0.593–0.973]; interrater: 0.794 [95%CI, 0.159–0.949]).

**Table 3:** Echocardiographic parameters at study time t_0_ and t_1_ presented as means and standard deviation or median and IQR.

		*t*_0_ ^a^		*t*_1_ ^b^	*p*-value^#^
	Means ± SD	Median (IQR)	Means ± SD	Median (IQR)	
HR (bpm)	64.09 ± 10.15	63.50 (57.5–69.25)	63.02 ± 11.15	62.50 (54.75–70)	0.290
Systolic BP, mmHg	118.68 ± 12.78	120 (110–125)	120.47 ± 14.49	120 (110–125)	0.540
Diastolic BP, mmHg	77.14 ± 8.92	80 (70–80)	77.47 ± 9.59	80 (70–80)	0.885
LVIDd (mm)	50.24 ± 4.64	49.50 (47.30–53.2)	49.63 ± 5.13	49.60 (45.85–53.65)	0.147
LVIDd/BSA	26.71 ± 2.36	26.65 (25.01–28.14)	26.43 ± 2.40	26.44 (24.82–28.02)	0.220
LVIDs (mm)	31.95 ± 4.76	31.85 (28.7–35.38)	31.85 ± 5.20	30.90 (28.7–34.3)	0.492
LVIDs/BSA	16.97 ± 2.35	16.99 (15.77–18.45)	16.95 ± 2.35	16.82 (15.47–18.56)	0.667
LV EF (%)	70.62 ± 7.73	71.35 (66.33–77.3)	69.66 ± 10.87	71.10 (65.6–77.15)	0.864
FS (%)	36.61 ± 6.24	35.75 (32.3–39.95)	35.47 ± 7.32	36 (30.5–40.7)	0.487
LV mass (g)	155.21 ± 47.99	146.50 (120.5–182.75)	154.54 ± 46.33	149.50 (119.5–179)	0.554
LV mass/BSA (g/m^2^)	81.25 ± 18.52	79.86 (67.12–91.89)	81.32 ± 17.93	81.65 (66.38–92.19)	0.717
IVSd (mm)	8.63 ± 1.48	8.65 (7.7–9.38)	8.76 ± 1.26	8.90 (8–9.6)	0.259
LVPWd (mm)	8.72 ± 1.56	8.60 (7.4–9.8)	8.76 ± 1.49	8.60 (7.85–9.7)	0.693
SV (ml)	92.99 ± 25.89	89.10 (78.50–115)	89.82 ± 27.43	87.35 (71.18–110.25)	0.241
LV GLS (%)	−18.82 ± 2.02	−18.86 (−19.87 to −17.30)	−19.46 ± 2.05	−19.52 (−20.67 to −18.28)	<.001***
TAPSE (mm)	25.64 ± 4.84	24.55 (22.38–28.5)	26.12 ± 4.18	25.60 (23.4–27.7)	0.247
E/A	1.45 ± 0.43	1.40 (1.15–1.7)	1.46 ± 0.36	1.40 (1.2–1.7)	0.717
E/Él	5.79 ± 1.72	5.40 (4.8–6.7)	6.17 ± 5.29	5.40 (4.5–6.7)	0.662
E/Ém	7.87 ± 2.43	7.25 (6.38–8.7)	7.94 ± 2.58	7.40 (6.3–9.2)	0.839
DecTime (ms)	173.42 ± 56.47	164 (129–209)	173.66 ± 50.21	166 (137–202)	0.775

*t*_0_, first clinical evaluation; *t*_1_, second clinical evaluation; SD, standard deviation; IQR, interquartile range; HR, heart rate; Bpm, beats per minute; BP, blood pressure; LVIDd, left ventricular internal diameter end diastole; LVIDd/BSA, left ventricular internal diameter end diastole/body surface area; LVIDs, left ventricular internal diameter end systole; LVIDs/BSA, left ventricular internal diameter end systole/body surface area; LV EF, left ventricular ejection fraction by Simpson; FS, fractional shortening; LV mass, left ventricular mass; LV mass/BSA, left ventricular mass/body surface area; IVSd, interventricular septal end diastole; LVPWd, left ventricular posterior wall end diastole; SV, stroke volume; LV GLS, left ventricular longitudinal strain; TAPSE, tricuspid annular plane systolic excursion. E/A ratio. E/Él ratio. E/Ém ratio. Dec Time, Deceleration Time. Significant results were presented as follows: *** < 0.001.

^a^ 96 athletes.

^b^ 96 athletes.

^#^ *P*-values from robust linear mixed-effects regression analysis (see Supplementary Table S1).

### 3.3. Cardiopulmonary exercise testing (CPET)

Maximum power was significant higher at *t*_1_ than at *t*_0_ (*t*_0_ 283.17 ± 83.20 Watt vs. *t*_1_ 286.24 ± 85.22 Watt, *p* = 0.009). There were no significant differences between *t*_0_ and *t*_1_ in performance parameters such as peak oxygen pulse or VE/VCO_2_ slope but there was a trend toward increased peak oxygen uptake (*t*_0_ 36.82 ± 11.14 ml/min/kg vs. *t*_1_ 38.68 ± 10.26 ml/min/kg, *p* = 0.069) (Table 4, Supplementary Table S1). Baseline heart rate was 64.09 ± 10.15 bpm at *t*_0_ and 63.02 ± 11.15 bpm at *t*_1_ and was not significantly different. Heart rate increased only slightly but not significantly (*t*_0_ 170.70 ± 17.50 bpm vs. *t*_1_ 172.63 ± 14.10 bpm, *p* = 0.480). Athletes achieved the same maximal effort at both time points (RER 1.22 vs. 1.22, *p* = 0.424) (Table 4).

**Table 4:** CPET parameters at study time t_0_ and t_1_ presented as means and standard deviation or median and IQR.

	*t*_0_		*t*_1_		*p*-value^a^
	Means ± SD	Median (IQR)	Means ± SD	Median (IQR)	
Maximum Power (Watt)	283.17 ± 83.20	289 (216–350)	286.24 ± 85.22	288.50 (224.5–250)	0.009**
Predicted maximum Power (%)^b^	160.65 ± 38.04	158.50 (136.75–180.5)	163.04 ± 37.56	161 (139.25–181.75)	0.099
VO_2_@VT1/kg (ml/min/kg)	22.58 ± 6.91	22.15 (17.68–26.68)	22.66 ± 7.28	22 (18.05–27.82)	0.615
Peak VO_2_/kg (ml/min/kg)	36.82 ± 11.14	37.80 (29.80–44.2)	38.68 ± 10.26	38.65 (30.95–45.88)	0.069
Peak RER	1.22 ± 0.08	1.22 (1.18–1.28)	1.22 ± 0.08	1.21 (1.17–1.26)	0.424
HR@VT1 (bpm)	125.40 ± 16.88	124 (113–136)	125.65 ± 17.13	125.50 (115.5–138.25)	0.678
HR@peak VO_2_ (bpm)	170.70 ± 17.50	174 (165.5–180.5)	172.63 ± 14.10	174.50 (166–182)	0.480
Predicted HR@peak VO_2_ (%)^b^	93.95 ± 9.01	95 (88.5–99)	95.02 ± 7.07	96 (90.25–99)	0.557
Peak Oxygen pulse (ml/beat)	16.27 ± 5.06	16.15 (12.73–19.65)	16.29 ± 4.79	16.80 (12.15–19.90)	0.327
Predicted peak Oxygen pulse (%)^b^	117.39 ± 25.13	116 (103.5–129.5)	118.56 ± 25.73	115 (102.25–130.75)	0.666
VE/VCO_2_ slope	25.20 ± 4.11	24.95 (22.45–27.53)	24.70 ± 3.35	24.70 (22.4–27.15)	0.203

*t*_0_, first clinical evaluation; *t*_1_, second clinical evaluation; SD, standard deviation; VO_2_@VT1/kg, respiratory oxygen uptake at first ventilatory threshold; Peak VO_2_/kg, peak respiratory oxygen uptake; Predicted VO_2_, predicted peak respiratory oxygen uptake; Peak RER, Respiratory Exchange Rate; HR@VT1, heart rate at first ventilatory threshold; HR@peak VO_2_, heart rate at peak respiratory oxygen uptake; Predicted HR@peak VO_2_, predicted heart rate at peak respiratory oxygen uptake; VE/VCO_2_ slope, ventilation/volume of CO_2_ slope. Significant results were presented as follows: ** < 0.01.

^a^ *P*-values from robust linear mixed-effects regression analysis (see Supplementary Table S1).

^b^ Calculated.

### 3.4. Correlation of LV GLS with performance parameters

LV GLS correlates inversely with RER (−0.316 [−0.501; 0.102], S = 129,938.15, *p* = 0.027) (Supplementary Table S3). This means that athletes with inadequate exercise intolerance also have the worse (more positive) GLS values. LV GLS did not correlate with other performance parameters such as oxygen uptake, peak oxygen pulse or VE/VCO_2_ slope (Supplementary Table S3).

### 3.5. Subgroup analysis of athletes with reduced LV GLS

**Figure 1:**
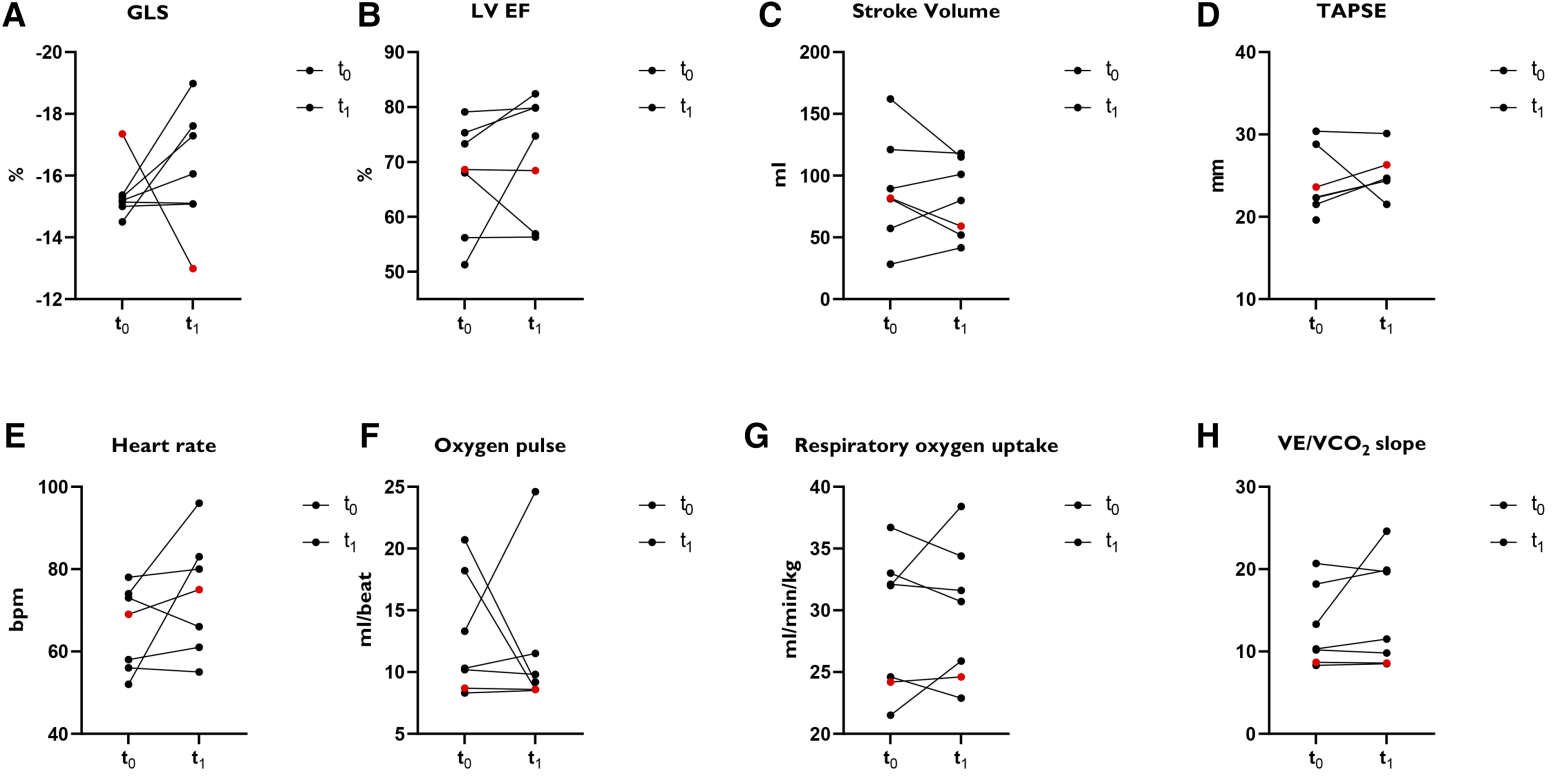
Subanalysis of athletes (*N* = 7) with LV GLS ≥ −16.0%. (**A**) GLS: Global longitudinal strain. (**B**) LV EF: left ventricular ejection fraction. (**C**) Stroke volume. (**D**) TAPSE: tricuspid annular plane systolic excursion. (**E**) Heart rate. Bpm = beats per minute. (**F**) Oxygen pulse. (**G**) Oxygen uptake. (**H**) VE/VO_2_ slope. Red points: Female athlete with a normal classified GLS at t_0_ and increased GLS at t_1_ without signs of heart disease or performance impairment.

Six athletes (mean age 37 years, three male) had a reduced LV GLS of ≥ −16.0% (less negative) (Figure 1). In four athletes the LV GLS normalized and in two athletes it remained constantly slightly reduced. Subacute myocarditis was detected by MRI in a female athlete with reduced LV GLS. LV EF improved here (51.3% vs. 74%), and performance was identical at both examination time points. An increase in peak VO_2_ was seen in two cases. In three cases it remained the same and in one athlete there was a decrease in peak VO_2_. In one female athlete (red points in Figure 1) out of 96 studied, a LV GLS previously classified as normal, increased without evidence of heart disease or performance impairment. For all parameters shown in Figure 1, there was no significant difference between the two study time points.

## 4. Discussion

Whether a reduction in LV GLS also leads to reduced performance in athletes after SARS-CoV-2 infection has been insufficiently investigated. We observed significant improvement in LV GLS approximately five months after COVID-19 compared to baseline at a median of two months after COVID-19, while we did not find any correlation between LV GLS and performance parameters, with the exception of RER.

### 4.1. Development of LV GLS

To our knowledge, this is the first study to investigate the course of LV GLS and the impact on performance in athletes after COVID-19. Our preliminary study results indicate a mild myocardial involvement in the form of reduced LV GLS in athletes after COVID-19 compared with healthy athletes (26). However, to date, there are no clear results and evidence that cardiac involvement occurs in athletes after COVID-19, and long-term observations in athletes are scarce (24, 25). Hospitalized patients without athletic background did not show significant improvement in LV-GLS at two months (27) or three months (28) or after acute infection: In the study by Baruch et al., 80 hospitalized patients (mean age 57.7 ± 14.9 years, 54 male) showed no significant difference in LV GLS 88.2 ± 33 days after baseline assessment, but 20 patients (25%) still had abnormal LV GLS (28). Fifty-two patients had moderate disease (pneumonia with a grade of ≥94% without oxygenation) and 28 patients had a severe disease course, so it is obvious that the included study population does not correspond to that of athletes in our work. In addition, the patients were significantly older and had previous cardiopulmonary diseases. Obesity, which may affect left ventricular function as measured by LV EF and LV GLS, was present in 19 patients (23.8%) (40–42).

Similar results were seen in the Danish prospective longitudinal cohort study ECHOVID-19 by Lassen et al. (27). Whereas right ventricular function improved after a median of 77 days, in contrast, LV GLS did not improve significantly. In addition, recovered COVID-19 patients had significantly lower LV GLS compared with age- and sex-matched controls from individuals participating in the Copenhagen City Heart Study (43). These 91 patients (mean age 63 ± 12 years, 54 male) were elderly and had heart failure or ischemic heart disease (11%) and hypertension (48%) and, most importantly, were also not athletes. LV GLS has been shown to be significantly reduced in patients with hypertension compared with normotensive control subjects (44) because blood pressure correlates with LV GLS (45, 46).

The findings of Baruch et al. and Lassen et al. lead in a different direction than our results, but this may be due to differences in the population studied (age, preexisting conditions, disease course). Patients who required hospitalization may have a higher prevalence of undetected subclinical heart disease than COVID-19 patients who were not hospitalized. Prolonged follow-up examinations may be needed here to show possible recovery. However, in the follow-up study by Karagodin et al., improvements in LV GLS were noted in patients with impaired baseline function (29). Overall, in this study, there was no significant change in LV GLS over time (230 ± 115 days) in 153 hospitalized patients (median age 57, 80 male). The improvement in LV GLS in patients with impaired baseline function may reflect recovery from acute myocardial injury in severely ill patients (31% in intensive care unit, 16.3% with ventilation, 8.5% with hemodynamic support). Our non-hospitalized athletes described mild courses with standard clinical symptoms of viral infection such as fever, cough, rhinorrhea, sore throat, dyspnea at rest or on exertion, and subjectively perceived decrease in performance (4). Cardiac symptoms such as palpitations, chest pain, and increased resting heart rate occurred in only 21%–26%. Recovery appears to be faster in healthy subjects without pre-existing myocardial dysfunction, which may be reflected in improvement of LV GLS. In general, highly trained athletes are known to have normal, albeit slightly lower, LV GLS and strain rate parameters compared to untrained control subjects (47, 48). Thus, there seems not to be a difference in baseline LV GLS here with that in the long-term studies presented. It still remains unknown what are the origins of the decreased LV myocardial function. It may be a consequence of direct cardiac injury from SARS-CoV-2 infection or a secondary consequence of systemic inflammation, or a combination of both (1, 3). Of course, ischemic injury caused by cardiac microvascular dysfunction cannot be excluded (49).

### 4.2. LV GLS and performance parameters

To date, no study has examined the relationship between LV GLS and physical performance in athletes recovering from COVID-19. However, in patients with ischemic heart disease (IHD), LV GLS is associated with decreased maximal oxygen uptake, which is an independent risk factor for adverse cardiovascular events (50). In addition, LV GLS correlated independently with peak VO_2_ in studies of patients with reduced (rEF) and pEF and was superior to LV EF in identifying patients with reduced exercise capacity (51–53).

In a study by Shimoni et al, patients were examined 57 (27–100) days after COVID-19. Subclinical impairment of LV function was shown to correlate with lower physical performance and duration, but no direct cause-effect relationship could be demonstrated (54). The correlation of LV GLS and physical performance could be due to persistent myocardial damage, and perhaps also to significant age differences (mean age 48 ± 12 years, 87 men) compared with our study group. In our study population of atheltes, LV GLS was within the normal range at *t*_0_ and *t*_1_, and we assume normal cardiac function with only mild myocardial involvement. Therefore, without the exception of RER, we could not demonstrate a correlation with performance parameters. The inverse correlation of GLS with RER indicates insufficient load intolerance at lower GLS values. There is no study to date that reflects this fact. However, in previous studies, submaximal exercise was observed in severe initial disease course (55) or as a sign of possible deconditioning (56) in Post-COVID patients. The number of athletes with reduced LV GLS (≥ −16.0%) may be too small to demonstrate statistical significance. Whether LV GLS correlates independently of COVID-19 with performance parameters in athletes in general has not been investigated to date. However, this could provide interesting additional information about cardiac adaptation processes in competitive as well as recreational athletes and could be integrated into diagnostics of the annual sports medical screening or return-to-sport examination in the future.

### 4.3. Development of spiroergometric parameters

Maximum power increased significantly from *t*_0 to_ *t*_1_ and there is a trend toward increased peak oxygen uptake. It cannot be ruled out that a break in training in the context of SARS-CoV-2 infection is causing poorer performance, which subsequently improves during the course. Improvement in performance could also be explained by the athletes' increasing recovery and predominantly being symptom-free at t_1_. The oxygen pulse, reflecting the maximal aerobic capacity, does not change significantly and heart rate increases only slightly but not significantly. These results emphasize that cardiac function does not appear to be impaired. Athletes achieved the same maximal effort at both time points (RER 1.22 vs. 1.22, *p* = 0.424). Similarly, in subgroup analysis of six athletes with probable myocardial dysfunction (GLS ≥ −16.0%), heart rate, LV GLS, peak oxygen pulse, or maximal oxygen uptake did not differ significantly between study time points.

This is broadly consistent with results from Komici et al, who found no decreased physical performance or impairment in pulmonary and cardiovascular function during the early recovery period (10–30 days) after COVID-19 in 24 competitive soccer players (mean age 23.5 years, 24 male) compared to healthy control athletes. However, LV GLS was not examined here (57). Two previously published studies showed an improvement in symptoms after COVID with corresponding improvements in CPET parameters over a longer observation period (58, 59). The main difference between the study by Moulson et al. and ours was that it included athletes with persistent cardiopulmonary symptoms, thus presenting a highly selected subgroup of athletes after COVID-19. These athletes were significantly more likely to report symptoms of dyspnea (76% vs. 21%), exercise intolerance (76% vs. 39%), chest pain (71% vs. 21%), and palpitations (57% vs. 21%) than the athletes we studied (who had partially recovered and were symptom-free), which may explain initially worse performance and subsequent improvement (58). Due to the different study populations and designs, there are no consensus data available so far, which limits the comparability between current studies.

### 4.4. Strengths and limitations

This presented study is limited by the longitudinal design, as strain values and performance parameters of athletes from periods prior to COVID-19 are not available. Follow-up examinations were performed after five months on the assumption that this period was sufficiently long to detect recovery of cardiac function. For possible variables influencing GLS, such as age, sex, heart rate, BMI, systolic or diastolic blood pressure, we performed multivariate linear regression. Significant changes in LV GLS persisted over the observation period. Although the LV GLS determination can be software and investigator experience dependent, our results show high intrarater and interrater reliability. On the one hand, the clearly defined population of study participants, consisting of athletes, limits the generalizability of the results to the general population. But, on the other hand, it allows an assessment of a specific group. It should also be emphasized that we achieved a meaningful case number of athletes for a single-centre study. Finally, although an association between COVID-19 and the occurrence of pathological examination findings up to myocarditis is suggested, direct evidence is still lacking.

### 4.5. Conclusion

We observed significant improvements in LV GLS approximately five months after COVID-19 compared with baseline at a median of two months after COVID-19. Therefore, we assume that the significant LV-GLS differences reflect possible mild myocardial involvement during or shortly after COVID-19. Except for RER, we did not find a correlation between LV GLS and performance parameters. This could indicate that mild cardiac dysfunction in athletes with mild disease course does not necessarily contribute to decreased performance after COVID-19. However, an inverse correlation between GLS and RER seems to indicate insufficient load intolerance at lower GLS values. Here, further studies on the development of GLS in athletes or in the general population with moderate and severe disease courses would be informative as well as the comparison of pre-COVID-19 with post-COVID-19 echocardiography to evaluate the effects of COVID-19 on cardiac function.

## Data Availability

The original contributions presented in the study are included in the article/Supplementary Material, further inquiries can be directed to the corresponding author.
