# Supplementary material for: Assessment of myocardial function and cardiac performance using left ventricular global longitudinal strain in athletes after COVID-19: a follow-up study
Source: Front Cardiovasc Med. 2023 Oct 9;10:1240278. doi: 10.3389/fcvm.2023.1240278 (PMC10591089; doi:10.3389/fcvm.2023.1240278)
Supplement: Supplementary file 1 [file Table1.docx]

Supplement:

**Table S1. Robust mixed-linear model on changes in studied variables between study time points.**

|  | **Parameter** | **Coefficient** | **SE** | **95% CI** | **t** | **p** |
| --- | --- | --- | --- | --- | --- | --- |
| HR | (Intercept) | 63.72 | 1.06 | [61.65, 65.79] | 60.22 | < .001 |
|  | Time | -1.07 | 1.01 | [-3.04, 0.91] | -1.06 | 0.290 |
| ᵟ patient  ᵟ residual | 7.48  6.80 |  |  |  |  |  |
| Systolic BP | (Intercept) | 118.63 | 1.11 | [116.46, 120.81] | 106.80 | < .001 |
|  | Time | 0.86 | 1.39 | [-1.88, 3.59] | 0.61 | 0.540 |
| ᵟ patient  ᵟ residual | 5.48  9.03 |  |  |  |  |  |
| Diastolic BP | (Intercept) | 77.23 | 0.96 | [75.35, 79.11] | 80.52 | < .001 |
|  | Time | -0.20 | 1.40 | [-2.94, 2.53] | -0.14 | 0.885 |
| ᵟ patient  ᵟ residual | 0.00  9.12 |  |  |  |  |  |
| LVIDd | (Intercept)  Time | 50.13  -0.54 | 0.52  0.37 | [49.11, 51.15]  [-1.28, 0.19] | 96.12  -0.69 | < .001 |
| ᵟ patient  ᵟ residual | 4.30  2.52 |  |  |  |  |  |
| LVIDd/BSA | (Intercept)  Time | 26.70  -0.25 | 0.24  0.20 | [26.23, 27.16]  [-0.64, 0.15] | 112.22  -1.23 | < .001  0.220 |
| ᵟ patient  ᵟ residual |  |  |  |  |  |  |
| LVIDs  ᵟ patient  ᵟ residual | (Intercept)  Time  3.48 | 32.02  -0.34 | 0.50  0.49 | [31.04, 33.00]  [-1.30, 0.62] | 63.85  -0.69 | < .001  0.492 |
| LVIDs/BSA  ᵟ patient  ᵟ residual | (Intercept)  Time | 17.07  -0.12 | 0.24  0.28 | [16.60, 17.53]  [-0.67, 0.43] | 72.35  -0.43 | < .001  0.667 |
| LV EF | (Intercept) | 71.09 | 0.87 | [69.38, 72.80] | 81.61 | < .001 |
|  | Time | -0.21 | 1.24 | [-2.63, 2.21] | -0.17 | 0.864 |
| ᵟ patient  ᵟ residual | 0.00  8.32 |  |  |  |  |  |
| FS | (Intercept) | 36.27 | 0.68 | (34.93, 37.61] | 53.07 | < .001 |
|  | Time | -0.67 | 0.97 | [-2.57, 1.23] | -0.70 | 0.487 |
| ᵟ patient  ᵟ residual | 0.00  6.53 |  |  |  |  |  |
| LV mass | (Intercept) | 153.95 | 4.99 | [144.16, 163.73] | 30.84 | < .001 |
|  | Time | -1.78 | 3.01 | [-7.67, 4.11] | -0.59 | 0.554 |
| ᵟ patient  ᵟ residual | 43.24  19.71 |  |  |  |  |  |
| LV mass/BSA | (Intercept) | 80.85 | 1.95 | [77.04, 84.67] | 41.55 | < .001 |
|  | Time | -0.58 | 1.59 | [-3.70, 2.54] | -0.36 | 0.717 |
| ᵟ patient  ᵟ residual | 15.31  10.37 |  |  |  |  |  |
| IVSd | (Intercept) | 8.63 | 0.13 | [8.37, 8.89] | 65.06 | < .001 |
|  | Time | 0.15 | 0.14 | [-0.11, 0.42] | 1.13 | 0.259 |
| ᵟ patient  ᵟ residual | 0.87  0.92 |  |  |  |  |  |
| LVPWd | (Intercept) | 8.67 | 0.16 | [8.37, 8.98] | 55.36 | < .001 |
|  | Time | 0.07 | 0.17 | [-0.27, 0.40] | 0.40 | 0.693 |
| ᵟ patient  ᵟ residual | 0.97  1.14 |  |  |  |  |  |
| SV | (Intercept) | 92.17 | 2.87 | [86.53, 97.80] | 32.06 | < .001 |
|  | Time | -3.03 | 2.58 | [-8.08, 2.03] | -1.17 | 0.241 |
| ᵟ patient  ᵟ residual | 21.31  16.60 |  |  |  |  |  |
| LV GLS | (Intercept) | -18.81 | 0.21 | [-19.21, -18.41] | -91.69 | < .001 |
|  | Time | -0.61 | 0.18 | [-0.97, -0.26] | -3.38 | < .001*** |
| ᵟ patient  ᵟ residual | 1.52  1.22 |  |  |  |  |  |
| TAPSE | (Intercept) | 25.26 | 0.44 | [24.41, 26.12] | 57.72 | < .001 |
|  | Time | 0.54 | 0.47 | [-0.38, 1.47] | 1.16 | 0.247 |
| ᵟ patient  ᵟ residual | 2.71  3.09 |  |  |  |  |  |
| E/A | (Intercept) | 1.43 | 0.04 | [1.35, 1.51] | 35.06 | < .001 |
|  | Time | 0.02 | 0.04 | [-0.07, 0.10] | 0.36 | 0.717 |
| ᵟ patient  ᵟ residual | 0.26  0.29 |  |  |  |  |  |
| E/E´l | (Intercept) | 5.67 | 0.17 | [5.33, 6.01] | 32.47 | < .001 |
|  | Time | -0.11 | 0.25 | [-0.60, 0.38] | -0.44 | 0.662 |
| ᵟ patient  ᵟ residual | 0.00  1.61 |  |  |  |  |  |
| E/E´m | (Intercept) | 7.67 | 0.22 | [7.25, 8.10] | 35.46 | < .001 |
|  | Time | 0.05 | 0.24 | [-0.43, 0.53] | 0.20 | 0.839 |
| ᵟ patient  ᵟ residual | 1.28  1.58 |  |  |  |  |  |
| Dect Time | (Intercept) | 168.55 | 6.39 | [156.02, 181.07] | 26.38 | < .001 |
|  | Time | 2.60 | 9.10 | [-15.25, 20.44] | 0.29 | 0.775 |
| ᵟ patient  ᵟ residual | 0.00  51.75 |  |  |  |  |  |
| Maximum Power | (Intercept) | 283.11 | 9.03 | [265.42, 300.81] | 31.36 | < .001 |
|  | Time | 9.11 | 3.51 | [2.23, 15.99] | 2.60 | 0.009** |
| ᵟ patient  ᵟ residual | 80.48  22.07 |  |  |  |  |  |
| Predicted maximum Power | (Intercept) | 157.88 | 3.65 | [150.73, 165.02] | 43.30 | < .001 |
|  | Time | 3.69 | 2.23 | [-0.69, 8.06] | 1.65 | 0.099 |
| ᵟ patient  ᵟ residual | 30.33  13.60 |  |  |  |  |  |
| VO_2_@VT1/kg | (Intercept) | 60.83 | 1.21 | [58.45, 63.21] | 50.14 | < .001 |
|  | Time | 0.87 | 1.74 | [-2.53, 4.27] | 0.50 | 0.615 |
| ᵟ patient  ᵟ residual | 0.00  11.10 |  |  |  |  |  |
| Peak VO_2_/kg | (Intercept) | 37.11 | 1.06 | [35.03, 39.18] | 34.99 | < .001 |
|  | Time | 1.15 | 0.63 | [-0.09, 2.38] | 1.82 | 0.069 |
| ᵟ patient  ᵟ residual | 8.97  3.93 |  |  |  |  |  |
| Peak RER | (Intercept) | 1.23 | 0.008 | [1.21, 1.24] | 137.14 | < .001 |
|  | Time | -0.01 | 0.01 | [-0.04, 0.01] | -0.80 | 0.424 |
| ᵟ patient  ᵟ residual | 0.00  0.08 |  |  |  |  |  |
| HR@VT1 | (Intercept) | 124.90 | 1.85 | [121.28, 128.52] | 67.58 | < .001 |
|  | Time | 0.72 | 1.73 | [-2.68, 4.12] | 0.42 | 0.678 |
| ᵟ patient  ᵟ residual | 12.90  10.49 |  |  |  |  |  |
| Predicted HR@VT1 | (Intercept) | 72.28 | 0.93 | [70.46, 74.10] | 77.79 | < .001 |
|  | Time | -0.08 | 1.18 | [-2.39, 2.24] | -0.07 | 0.947 |
| ᵟ patient  ᵟ residual | 3.94  7.27 |  |  |  |  |  |
| HR@peak VO_2_ | (Intercept) | 173.14 | 1.39 | [170.42, 175.87] | 124.53 | < .001 |
|  | Time | 0.91 | 1.29 | [-1.62, 3.44] | 0.71 | 0.480 |
| ᵟ patient  ᵟ residual | 9.71  7.88 |  |  |  |  |  |
| Predicted HR@peak VO_2_ | (Intercept) | 94.59 | 0.80 | [93.03, 96.15] | 118.74 | < .001 |
|  | Time | 0.38 | 0.65 | [-0.89, 1.65] | 0.59 | 0.557 |
| ᵟ patient  ᵟ residual | 6.02  3.95 |  |  |  |  |  |
| Peak Oxygen pulse | (Intercept) | 16.08 | 0.51 | [15.09, 17.08] | 31.65 | < .001 |
|  | Time | 0.27 | 0.28 | [-0.27, 0.81] | 0.98 | 0.327 |
| ᵟ patient  ᵟ residual | 4.36  1.72 |  |  |  |  |  |
| Predicted peak Oxygen pulse | (Intercept) | 115.90 | 2.34 | [111.31, 120.49] | 49.48 | < .001 |
|  | Time | 0.87 | 2.01 | [-3.07, 4.81] | 0.43 | 0.666 |
| ᵟ patient  ᵟ residual | 17.19  12.26 |  |  |  |  |  |
| VE/VCO_2_ slope | (Intercept) | 25.02 | 0.38 | [24.28, 25.77] | 65.99 | < .001 |
|  | Time | -0.42 | 0.33 | [-1.06, 0.22] | -1.27 | 0.203 |
| ᵟ patient  ᵟ residual | 2.79  2.01 |  |  |  |  |  |

Abbreviations: SE=standard error, CI=confidence interval, HR=heart rate. BP= blood pressure, LVIDd=left ventricular internal diameter end diastole. LVIDd/BSA=left ventricular internal diameter end diastole/body surface area. LVIDs=left ventricular internal diameter end systole. LVIDs/BSA=left ventricular internal diameter end systole/body surface area. LV EF=left ventricular ejection fraction by Simpson. FS=fractional shortening. LV mass=left ventricular mass. LV mass/BSA=left ventricular mass/body surface area. IVSd=interventricular septal end diastole. LVPWd=left ventricular posterior wall end diastole. SV=stroke volume. LV GLS=left ventricular longitudinal strain. TAPSE=tricuspid annular plane systolic excursion. E/A ratio. E/E´l ratio. E/E´m ratio. Dec Time=Deceleration Time. CK=Creatine kinase. CRP=C-reactive protein. Peak RER=Respiratory Exchange Rate. Significant results were presented as follows: * < 0.05 ** < 0.01 *** < 0.001.

**Table S2: Multivariate linear regression on changes in GLS between study time points accounting for confounding variables.**

| **Parameter** | **Coefficient** | **SE** | **95% CI** | **t** | **p** |
| --- | --- | --- | --- | --- | --- |
| (Intercept) | -18.55 | 0.26 | [-19.05, -18.04] | -72.05 | < .001 |
| Time | -0.60 | 0.18 | [-0.95, -0.24] | -3.32 | < .001 |
| Sex | -0.59 | 0.36 | [-1.29, 0.11] | -1.65 | 0.098 |
| (Intercept) | -19.12 | 0.54 | [-20.17, -18.07] | -35.69 | < .001 |
| Time | -0.61 | 0.18 | [-0.97, -0.26] | -3.38 | < .001 |
| Age | 0.009 | 0.01 | [-0.02, 0.04] | 0.61 | 0.541 |
| (Intercept) | -22.22 | 1.16 | [-24.49, -19.95] | -19.16 | < .001 |
| Time | -0.64 | 0.19 | [-1.02, -0.26] | -3.30 | < .001 |
| BMI | 0.14 | 0.05 | [0.05, 0.24] | 3.00 | 0.003 |
| (Intercept) | -17.53 | 1.21 | [-19.89, -15.17] | -14.54 | < .001 |
| Time | -0.66 | 0.20 | [-1.05, -0.27] | -3.33 | < .001 |
| Systolic BP | -0.01 | 0.01 | [-0.03, 0.009] | -1.05 | 0.294 |
| (Intercept) | -18.78 | 1.09 | [-20.92, -16.64] | -17.18 | < .001 |
| Time | -0.68 | 0.20 | [-1.08, -0.29] | -3.39 | < .001 |
| Diastolic BP | 0.000 | 0.01 | [-0.03, 0.03] | 0.004 | 0.997 |
| (Intercept) | -19.44 | 0.86 | [-21.13, -17.75] | -22.55 | < .001 |
| Time | -0.60 | 0.18 | [-0.96, -0.24] | -3.28 | < .001 |
| HR | 0.009 | 0.01 | [-0.02, 0.04] | 0.75 | 0.452 |

Abbreviations: SE=standard error, CI=confidence interval, BMI=body mass index, BP=blood pressure HR=heart rate. Significant results were presented as follows: * < 0.05 ** < 0.01 *** < 0.001.

**Table S3: Correlation analysis of GLS with performance and laboratory parameters.**

| **Parameter 1** | **Parameter 2** | **rho** | **CI** | **S** | **p** |
| --- | --- | --- | --- | --- | --- |
| **LV GLS** | Maximum Power | -0.148 | [-0.351; 0.068] | 134890.65 | 0.432 |
| **LV GLS** | Predicted maximum Power | -0.258 | [-0.452; -0.039] | 124211.49 | 0.100 |
| **LV GLS** | VO_2_@VT1/kg | -0.263 | [-0.453; -0.051] | 143451.68 | 0.079 |
| **LV GLS** | Peak VO_2_/kg | -0.169 | [-0.370; 0.047] | 137382.03 | 0.345 |
| **LV GLS** | RER | -0.316 | [-0.501; -0.102] | 129938.15 | 0.027* |
| **LV GLS** | HR@VT1 | -0.057 | [-0.276; 0.167] | 100752.41 | 0.854 |
| **LV GLS** | Predicted HR@VT1 | -0.113 | [-0.326; 0.112] | 106013.94 | 0.620 |
| **LV GLS** | HR@paek VO_2_ | 0.133 | [-0.092; 0.344] | 82634.52 | 0.516 |
| **LV GLS** | Predicted HR@peak VO_2_ | 0.218 | [-0.004; 0.420] | 74508.29 | 0.195 |
| **LV GLS** | Peak Oxygen pulse | -0.068 | [-0.280; 0.149] | 121334.60 | 0.781 |
| **LV GLS** | Predicted peak Oxygen pulse | -0.219 | [-0.420; 0.003] | 116123.20 | 0.195 |
| **LV GLS** | VE/VCO_2_ slope | 0.127 | [-0.096; 0.338] | 86199.40 | 0.539 |
| **LV GLS** | CK | -0.114 | [-0.315; 0.097] | 154156.17 | 0.573 |
| **LV GLS** | Troponin T | 0.069 | [-0.150; 0.281] | 102197.95 | 0.781 |
| **LV GLS** | CRP | 0.131 | [-0.080; 0.330] | 120324.60 | 0.499 |
| **LV GLS** | Ferritin | 0.076 | [-0.134; 0.280] | 127876.01 | 0.745 |
| **LV GLS** | Hemoglobin | 0.101 | [-0.111; 0.305] | 120484.37 | 0.632 |

Abbreviations: CI=confidence interval. LV GLS=left ventricular longitudinal strain. VO2@VT1/kg= oxygen uptake at first ventilatory threshold. Peak VO2/kg= peak oxygen uptake. RER=Respiratory Exchange Rate. HR@VT1= heart rate at first ventilatory threshold. Predicted HR@VT1=predicted heart rate at first ventilatory threshold. HR@peak VO_2_=heart rate at peak oxygen uptake. Predicted HR@peak VO_2_=predicted heart rate at peak oxygen uptake. VE/VCO2 slope= ventilation/volume of CO2 slope. CK=creatine kinase. CRP=C-reactive protein. Significant results were presented as follows: * < 0.05 ** < 0.01 *** < 0.001.
